# Avaliação do Fluxo e das Dimensões do Seio Coronário por Ecocardiografia Transtorácica em Pacientes com Taquicardia por Reentrada Nodal Atrioventricular

**DOI:** 10.36660/abc.20250891

**Published:** 2026-06-22

**Authors:** Ali Duygu, Alkame Akgümüş, Ahmet Balun, Fahri Er

**Affiliations:** 1 Bandirma Onyedi Eylul Universitesi Department of Cardiology Bandirma Turquia Bandirma Onyedi Eylul Universitesi – Department of Cardiology, Bandirma – Turquia

**Keywords:** Taquicardia por Reentrada no Nó Atrioventricular, Seio Coronário, Ecocardiografia Doppler

## Abstract

**Fundamento:**

O seio coronário (SC) desempenha um papel fundamental na drenagem venosa cardíaca e está anatomicamente adjacente à região nodal atrioventricular. Embora o aumento estrutural do SC já tenha sido descrito em pacientes com taquicardia por reentrada nodal atrioventricular (TRNAV), o fluxo do seio coronário (FSC) avaliado por ecocardiografia transtorácica (ETT) ainda não havia sido investigado nessa população.

**Objetivo:**

Avaliar a relação entre TRNAV e FSC.

**Métodos:**

Este estudo retrospectivo, de centro único, incluiu 35 pacientes com TRNAV submetidos à ablação bem-sucedida da via lenta e 34 controles saudáveis pareados por idade e sexo. Todos os participantes realizaram ecocardiografia transtorácica abrangente. O diâmetro do óstio do SC, o diâmetro do SC e o FSC foram medidos por Doppler pulsado. O FSC foi calculado utilizando parâmetros geométricos e derivados do Doppler. Valores de p < 0.05 foram considerados estatisticamente significativos.

**Resultados:**

Os parâmetros globais de função sistólica e diastólica dos ventrículos esquerdo e direito foram semelhantes entre os grupos. Pacientes com TRNAV apresentaram diâmetros significativamente maiores do óstio do SC e do SC em comparação aos controles (ambos p<0.001). Apesar desse aumento, o FSC foi significativamente reduzido no grupo TRNAV (460.8 ± 83.3 vs. 523.8 ± 90.1 mL/min, p=0.004), acompanhado por um menor integral velocidade-tempo do SC. Notavelmente, o FSC indexado à massa ventricular esquerda também permaneceu significativamente menor nos pacientes com TRNAV, indicando que a redução do fluxo foi independente da massa miocárdica.

**Conclusão:**

Pacientes com TRNAV apresentam um perfil hemodinâmico distinto do SC, caracterizado por aumento estrutural do SC e redução do FSC avaliado por ecocardiografia transtorácica. Esses achados sugerem alterações na hemodinâmica venosa coronária na TRNAV e fornecem novos insights fisiopatológicos por meio de uma modalidade de imagem não invasiva.

## Graphical abstract

**Figura Central:**
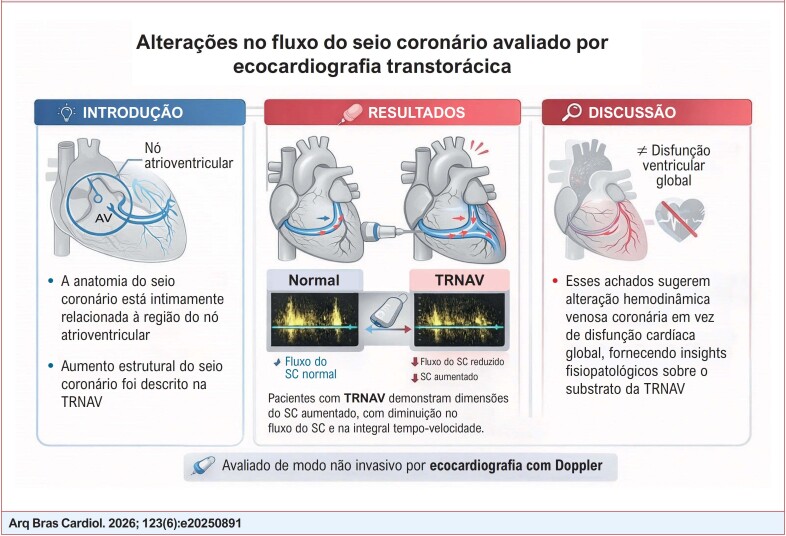
Avaliação do Fluxo e das Dimensões do Seio Coronário por Ecocardiografia Transtorácica em Pacientes com Taquicardia por Reentrada Nodal Atrioventricular

SC: seio coronário; TRNAV: taquicardia por reentrada nodal atrioventricular.

## Introdução

O seio coronariano (SC) é uma estrutura fundamental para a drenagem venosa cardíaca, conduzindo o sangue das veias cardíacas para o átrio direito. Devido à sua proximidade com o nó atrioventricular (AV), desempenha um papel crucial em procedimentos eletrofisiológicos e terapias de ablação. A taquicardia por reentrada nodal atrioventricular (TRNAV) é a forma mais comum de taquicardia supraventricular (TSV) paroxística. Ela é causada por um circuito de reentrada envolvendo vias duplas localizadas ao redor do nó AV, particularmente dentro do triângulo de Koch — uma área anatômica definida pelo óstio do SC (O-SC), pelo tendão de Todaro e pelo anel da valva tricúspide.^1^ Embora o mecanismo exato ainda não esteja totalmente esclarecido, estudos sugerem que a TRNAV está relacionada a um circuito de reentrada entre vias lenta e rápida situadas na região peri-O-SC.^2^ A ablação da TRNAV também é realizada próxima ao O-SC, dentro do triângulo de Koch. Estudos prévios demonstraram que o diâmetro do O-SC é maior em pacientes com TRNAV, possivelmente alterando vias de condução e contribuindo para a arritmogênese. No entanto, o papel do fluxo no seio coronariano (FSC), um índice mensurável de perfusão miocárdica, ainda não foi investigado no contexto da TRNAV.^3-5^

Neste estudo, buscamos explorar a associação entre TRNAV e o fluxo no seio coronariano utilizando ecocardiografia transtorácica (ETT).

## Método

O estudo foi conduzido com a aprovação do Comitê de Ética em Pesquisa Não Intervencional em Ciências da Saúde da Universidade Bandırma Onyedi Eylül (Data: 06.02.2025, Número: E-67961857-050.04-2500011721). Todos os procedimentos foram realizados de acordo com as diretrizes éticas e os princípios da Declaração de Helsinque.

### Desenho do estudo e população

Este foi um estudo retrospectivo, unicêntrico, que incluiu 35 pacientes consecutivos diagnosticados com TRNAV e 34 controles saudáveis pareados por idade e sexo. Todos os pacientes do grupo TRNAV foram submetidos à ablação bem-sucedida da via lenta.

Foram excluídos pacientes com disfunção sistólica do ventrículo esquerdo (VE), síndrome da apneia obstrutiva do sono, histórico de uso de medicamentos ou substâncias, insuficiência renal ou hepática crônica, fibrilação atrial, cardiopatia congênita, histórico de próteses valvares, diâmetro da raiz aórtica maior que 40 mm, valva aórtica bicúspide ou janela ecocardiográfica inadequada. Pacientes com histórico de cirurgia de revascularização miocárdica (CRM), síndrome coronariana aguda (SCA), acidente vascular cerebral isquêmico, hipertensão portal, idade inferior a 18 anos, doença do tecido conjuntivo, insuficiência ou estenose valvar avançada, insuficiência cardíaca direita, diagnóstico de hipertensão pulmonar ou histórico de embolia pulmonar também foram excluídos.

Dados demográficos, clínicos e laboratoriais foram obtidos dos prontuários hospitalares.

### Procedimento eletrofisiológico

Cateteres foram utilizados como dispositivos de acesso percutâneo e posicionados pela técnica de Seldinger, com fluoroscopia empregada para visualizar o avanço dos cateteres. Para a canulação do SC, foi utilizada abordagem inferior ou superior (cateter decapolar dirigível 6F, 2,52 mm, 110 cm, Boston Scientific, Massachusetts, EUA). Dois cateteres quadripolares foram posicionados no ventrículo direito e na região do feixe de His. Um sistema multicanal de registro eletrofisiológico foi utilizado para obter ECG de superfície de 12 derivações e eletrogramas intracardíacos.

Cada paciente com TSV foi submetido a um estudo eletrofisiológico padrão, incluindo testes de extraestímulos atriais e ventriculares, bem como *overdrive pacing*, para induzir a taquicardia e determinar seu mecanismo.^6^ O diagnóstico de TRNAV foi estabelecido de acordo com critérios padrão. Após a confirmação da TRNAV, realizou-se a modificação da via lenta sob orientação fluoroscópica e/ou mapeamento tridimensional. O triângulo inferior de Koch foi identificado como a região-alvo para a ablação da via lenta.

### Ecocardiografia

A ETT bidimensional (Vivid T8, GE Vingmed, EUA) foi utilizada para todas as medições. As avaliações ecocardiográficas foram realizadas com os pacientes em decúbito lateral esquerdo. A fração de ejeção do ventrículo esquerdo (FEVE) foi calculada pelo método de Simpson modificado. A espessura do septo interventricular (SIV), a espessura da parede posterior (PP), o diâmetro diastólico final do ventrículo esquerdo (DDFVE), o diâmetro sistólico final do ventrículo esquerdo (DSFVE) e as medidas do átrio esquerdo (AE) foram obtidos a partir da janela paraesternal em eixo longo (PLAX).

**Figura 1:**
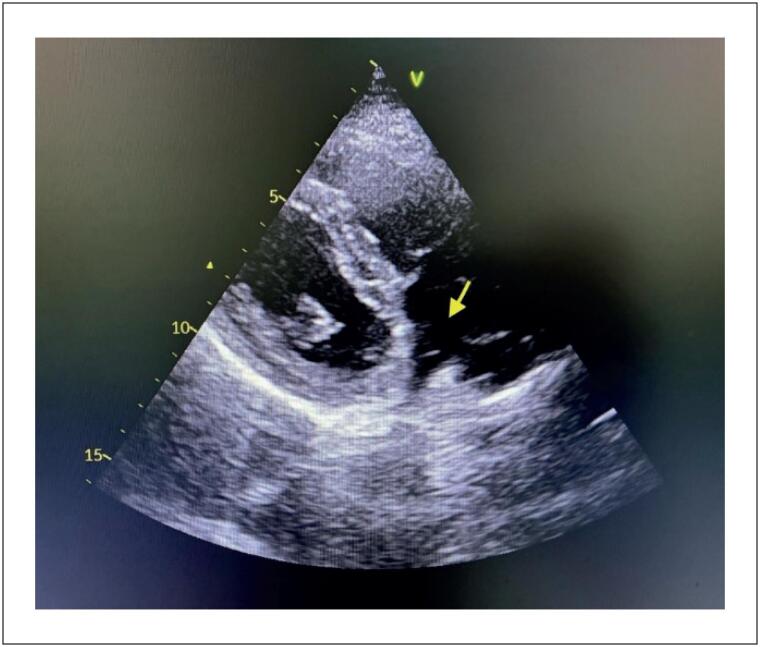
Visão do óstio do seio coronário.

O O-SC foi visualizado modificando-se as janelas PLAX e apical de quatro câmaras. As medições foram realizadas verticalmente a partir do ponto onde o O-SC se abre no átrio direito (Figura 1). As ondas E, A, E′ e A′ do fluxo mitral foram medidas por meio do Doppler tecidual (TDI), e a excursão sistólica do anel tricúspide (TAPSE) foi obtida com M-modo na janela apical de quatro câmaras.

**Figura 2:**
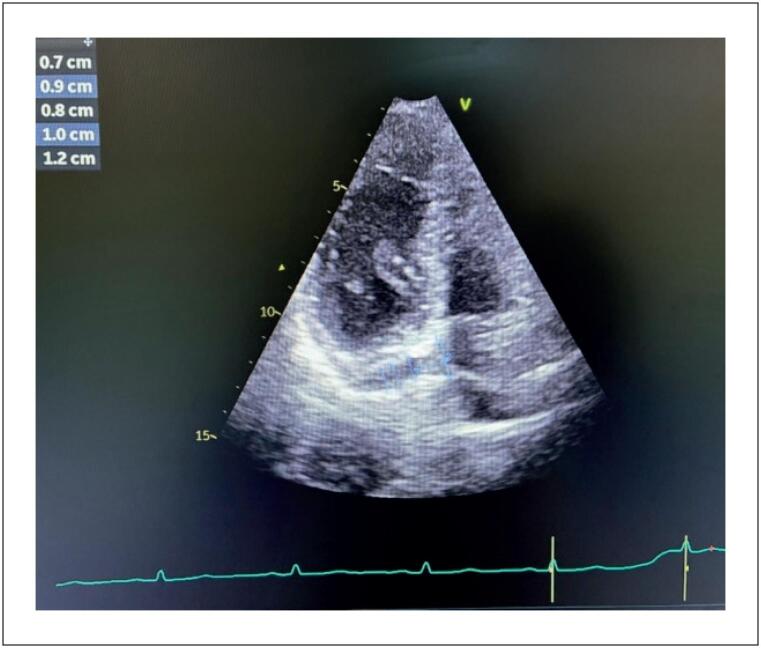
Medição do diâmetro do seio coronário.

**Figura 3:**
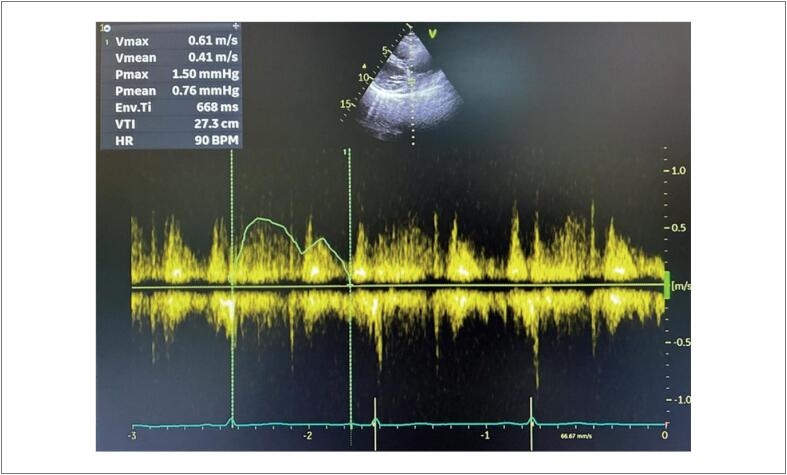
Medição do integral velocidade-tempo do seio coronário.

As janelas apicais de quatro câmaras e PLAX foram ajustadas para visualizar o SC. O d-SC foi medido em seu ponto mais amplo durante a sístole ventricular, mantendo-se uma distância de 1 cm do óstio (Figura 2). Para avaliar o FSC, o O-SC foi inicialmente identificado modificando-se a janela PLAX. O cursor do Doppler foi alinhado com o O-SC de modo que o ângulo entre o eixo do cursor e o eixo do SC fosse inferior a 30°. O volume de amostragem do Doppler pulsado (PW) foi então posicionado 3 mm dentro do óstio, em uma região minimamente afetada pelas contrações atriais. O integral velocidade-tempo (VTI) ventricular foi calculado traçando-se as ondas sistólica e diastólica do fluxo no SC (Figura 3). A frequência cardíaca foi registrada a partir do ECG no momento da aquisição das imagens.

O fluxo no seio coronariano (mL/min) foi calculado pela seguinte fórmula: $\left[\pi \times D^{2}/4\times VTI\times frequência\;cardíaca\right]$ . Aqui, *D* representa o diâmetro máximo do seio coronariano, e π é a razão entre a circunferência de um círculo e seu diâmetro.

### Análise estatística

O tamanho da amostra foi determinado por conveniência, incluindo todos os pacientes consecutivos que realizaram ablação de TRNAV durante o período do estudo e que atenderam aos critérios de inclusão. Um número igual de indivíduos controle foi incluído. As análises estatísticas foram realizadas utilizando o IBM SPSS Statistics versão 26. Variáveis categóricas são apresentadas como frequências absolutas e relativas, enquanto variáveis contínuas são expressas como média ± desvio-padrão para dados com distribuição normal ou como mediana e intervalo interquartil (IQR) para dados sem distribuição normal.

A normalidade da distribuição foi avaliada por métodos visuais (histogramas e gráficos de probabilidade) e por testes analíticos (teste de Shapiro–Wilk). As comparações entre dois grupos independentes foram realizadas utilizando o teste t de Student para variáveis com distribuição normal e o teste de Mann–Whitney U para variáveis sem distribuição normal. O teste do qui-quadrado foi utilizado para comparar variáveis categóricas. A significância estatística foi definida como p < 0,05.

## Resultados

Um total de 69 participantes foi incluído (35 pacientes com TRNAV e 34 controles). A idade média do grupo TRNAV foi de 32,00 ± 6,01 anos, enquanto a idade média do grupo controle foi de 29,35 ± 5,73 anos. As características demográficas e ecocardiográficas foram semelhantes entre os grupos, sem diferenças significativas na fração de ejeção do ventrículo esquerdo, dimensões das câmaras cardíacas ou valores de TAPSE.

Tanto o O-SC quanto o d-SC foram significativamente maiores no grupo TRNAV em comparação aos controles. O FSC e o VTI do SC foram significativamente menores no grupo TRNAV, e essas diferenças permaneceram significativas após indexação pela massa ventricular esquerda.

Todas as medidas ecocardiográficas estão resumidas na Tabela 1 e Tabela 2.

**Tabela 1:** Características demográficas e clínicas dos pacientes do estudo

		TRNAV	Controle	Valor p
**Idade (anos)**		32,00 ± 6,01	29,35 5,73	0,066^a^
**Gênero, n (%)**				
	Masculino	17 (48%)	14 (41%)	0,779^b^
	Feminino	18 (52%)	20 (59%)	
**Altura (cm)**		169,14 9,01	170,47 ± 8,70	0,536^a^
**Peso (kg)**		75,49 ± 10,74	71,53 ± 7,77	0,085^a^
**IMC (kg/m^2^)**		26,60 ± 4,73	24,68 ± 2,70	0,043^a^
**FC (beat/min)**		64,03 8,18	67,71 ± 8,39	0,070^a^

IMC: índice de massa corporal; FC: frequência cardíaca; TRNAV: taquicardia por reentrada nodal atrioventricular

a teste t de Student

b teste do qui-quadrado.

**Tabela 2:** Parâmetros ecocardiográficos

Variável	TRNAV	Controle	Valor p
**FEVE %**	60,97 ± 4,91	61,15 ± 4,45	0,877^a^
**TAPSE (mm)**	24,89 ± 3,11	26,26 ± 3,17	0,073^a^
**DDFVE (mm)**	47,14 ± 4,65	45,24 ± 4,23	0,080^a^
**DSFVE (mm)**	31,69 ± 4,23	29,79 ± 4,40	0,074^a^
**SIV (mm)**	11,20 ± 1,18	10,79 ± 0,67	0,087^a^
**PP (mm)**	10,51 ± 1,06	10,18 ± 0,67	0,122^a^
**Mit E (m/s)**	0,91 ± 0,06	0,91 ± 0,14	0,858^a^
**e’ (m/s)**	0,15 ± 0,01	0,14 ± 0,02	0,107^a^
**Mit A (m/s)**	0,68 ± 0,07	0,67 ± 0,10	0,502^a^
**a’ (m/s)**	0,08 ± 0,01	0,10 ± 0,03	0,336^a^
**E/e’**	5,98 ± 0,95	6,26 ± 1,26	0,298^a^
**Massa do VE (g)**	189,78 ± 46,20	144,41 ± 39,01	**0,000**^a^
**VTI (cm)**	12,33 ± 1,75	19,24 ± 2,60	**0,000**^a^
**O-SC (mm)**	1,19 ± 0,12	0,94 ± ,13	**0,000**^a^
**d-SC (mm)**	0,87 ± 0,08	0,72 ± 0,07	**0,000**^a^
**FSC (ml/min)**	460,75 ± 83,26	523,78 ± 90,06	**0,004**^a^
**Índice do FSC (mL/min/gram)**	0,371(0,277-0,539)	0,278(0,226-0,357)	**0,000**^b^

FEVE: fração de ejeção do ventrículo esquerdo; TAPSE: excursão sistólica do plano do anel tricúspide; DDFVE: diâmetro diastólico final do ventrículo esquerdo; DSFVE: diâmetro sistólico final do ventrículo esquerdo; SIV: septo interventricular; PP: parede posterior; VE: ventrículo esquerdo; VTI: integral velocidade-tempo ventricular; O-SC: óstio do seio coronário; d-SC: diâmetro do seio coronário; FSC: Fluxo do seio coronário

a Teste t de Student

b Teste U de Mann-Whitney.

## Discussão

Este estudo é o primeiro a investigar o FSC em pacientes com TRNAV utilizando ecocardiografia transtorácica. Demonstramos que o FSC é significativamente menor e que o d-SC é significativamente maior em pacientes com TRNAV em comparação com controles saudáveis. Essa redução do fluxo ocorre apesar da função sistólica e diastólica global preservada e é acompanhada por um aumento acentuado tanto do O-SC quanto do d-SC. Em conjunto, esses achados descrevem um fenótipo hemodinâmico venoso coronariano distinto em pacientes com TRNAV.

A TRNAV é a arritmia regular mais comum em humanos. Diversos modelos foram propostos para explicar seu mecanismo, considerando a anatomia complexa e as propriedades eletrofisiológicas do nó AV e das estruturas atriais adjacentes; no entanto, o circuito exato da TRNAV permanece incompletamente compreendido. Nos últimos anos, vários estudos buscaram esclarecer seu mecanismo subjacente. Um estudo examinou a relação entre o SC e a TRNAV e demonstrou que a ablação direcionada ao ponto de ativação atrial retrógrada mais precoce dentro do SC, em pacientes com ativação excêntrica do SC, resultou em desfechos altamente bem-sucedidos.^7,8^

Outro estudo comparou pacientes com TRNAV típica e atípica e encontrou um O-SC mais amplo nos pacientes com TRNAV típica.^9^ Em um estudo de Ezhumalai et al.,^10^ os diâmetros proximal e médio do SC foram avaliados em pacientes com TRNAV e TRAV utilizando ecocardiografia transesofágica, e observou-se que o SC era mais largo no grupo TRNAV. No entanto, Weiss et al.¹¹ mostraram que, embora o d-SC fosse maior em pacientes com TRNAV, não houve diferença significativa entre os grupos TRNAV e controle.

O aumento do O-SC estira o tecido periostial, aumentando a distância entre as vias lenta e rápida e levando a condução em direções diferentes.^8^ Além disso, um SC mais amplo pode predispor a diferentes padrões de fisiologia nodal ao separar as entradas atriais para o nó AV ou aumentar a condução anisotrópica.^5^

Uma observação fundamental do presente estudo é que o aumento do seio coronariano em pacientes com TRNAV não está associado a um aumento do fluxo no seio coronariano. Pelo contrário, o FSC e o integral velocidade-tempo do seio coronariano foram significativamente menores no grupo TRNAV. Essa combinação de maior diâmetro venoso e menor fluxo efetivo sugere um padrão de remodelamento venoso coronariano, e não simplesmente um aumento de fluxo. Tal remodelamento pode refletir alterações crônicas na anatomia atrial perinodal, na complacência venosa local ou no acoplamento átrio-venoso adjacente à região da via lenta, conforme previamente proposto em estudos eletrofisiológicos e anatômicos.^5,8^

É importante destacar que a estrutura e a função cardíacas globais estavam amplamente preservadas nos pacientes com TRNAV. A FEVE, os índices de enchimento diastólico e os parâmetros de função sistólica do ventrículo direito foram semelhantes entre os grupos, indicando que a redução do FSC não se deveu a disfunção ventricular evidente. Embora a massa ventricular esquerda tenha sido maior no grupo TRNAV, a indexação do FSC pela massa miocárdica não eliminou a diferença observada, reforçando a ideia de que o FSC reduzido representa uma característica hemodinâmica distinta, e não um efeito secundário da hipertrofia miocárdica.

O FSC já foi demonstrado como um marcador de perfusão miocárdica e circulação coronariana global em diversas condições clínicas, incluindo doença isquêmica do coração, miocardite, cardiomiopatias e após procedimentos de revascularização.^12-15^ Além disso, a redução do FSC tem sido associada a condições não coronarianas, como lesão miocárdica relacionada ao uso de substâncias.^16^ No entanto, seu papel em substratos arrítmicos não havia sido investigado. Os achados do presente estudo sugerem que alterações na hemodinâmica venosa coronariana podem coexistir com o substrato anatômico e eletrofisiológico da TRNAV. Embora não seja possível inferir causalidade a partir deste estudo observacional, o FSC reduzido pode refletir adaptações crônicas na região perinodal que contribuem para, ou resultam do, substrato da TRNAV.

Este estudo apresenta várias limitações. Seu delineamento retrospectivo e o tamanho amostral relativamente pequeno limitam a capacidade de estabelecer relações causais. O FSC foi avaliado em repouso e em um único momento, e medidas hemodinâmicas invasivas não estavam disponíveis para validação. Além disso, embora tenha sido realizado um alinhamento meticuloso do Doppler, a mensuração do FSC por ecocardiografia transtorácica permanece tecnicamente desafiadora. Ainda assim, a consistência dos achados entre múltiplos parâmetros relacionados ao seio coronariano reforça a validade das nossas observações.

## Conclusão

Pacientes com TRNAV apresentam um perfil hemodinâmico distinto do seio coronariano, caracterizado por aumento do seu diâmetro e redução do fluxo, conforme avaliado pela ecocardiografia transtorácica. Esses achados fornecem uma nova perspectiva não invasiva sobre a hemodinâmica venosa coronariana na TRNAV e sugerem que a avaliação do fluxo no seio coronariano pode servir como um parâmetro ecocardiográfico complementar para a compreensão da fisiopatologia dessa arritmia.

## Data Availability

Todo o conjunto de dados que dá suporte aos resultados deste estudo está disponível mediante solicitação ao autor correspondente.

## References

[1] Katritsis DG (2026). Atrioventricular Nodal Reentrant Tachycardia. Heart Rhythm.

[2] Katritsis DG, Marine JE, Contreras FM, Fujii A, Latchamsetty R, Siontis KC (2016). Catheter Ablation of Atypical Atrioventricular Nodal Reentrant Tachycardia. Circulation.

[3] Doig JC, Saito J, Harris L, Downar E (1995). Coronary Sinus Morphology in Patients with Atrioventricular Junctional Reentry Tachycardia and Other Supraventricular Tachyarrhythmias. Circulation.

[4] Ding L, Weng S, Zhai Z, Zhou B, Qi Y, Yu F (2022). Association between the Coronary Sinus Ostial Size and Atrioventricular Nodal Reentrant Tachycardia in Patients with Pulmonary Arterial Hypertension. Front Physiol.

[5] Leiria TLL, Branchi M, Sant'anna RT, Almeida ED, Pires LM, Kruse ML (2019). Coronary Sinus Cannulation Predicts Atrioventricular Nodal Reentry as Mechanism of Supraventricular Tachycardia. Indian Pacing Electrophysiol J.

[6] Issa ZF, Miller JM, Zipes DP (2019). Clinical Arrhythmology and Electrophysiology: A Companion to Braunwald's Heart Disease.

[7] Otomo K, Nagata Y, Uno K, Fujiwara H, Iesaka Y (2007). Atypical Atrioventricular Nodal Reentrant Tachycardia with Eccentric Coronary Sinus Activation: Electrophysiological Characteristics and Essential Effects of Left-Sided Ablation Inside the Coronary Sinus. Heart Rhythm.

[8] Jackman WM, Beckman KJ, McClelland JH, Wang X, Friday KJ, Roman CA (1992). Treatment of Supraventricular Tachycardia Due to Atrioventricular Nodal Reentry by Radiofrequency Catheter Ablation of Slow-Pathway Conduction. N Engl J Med.

[9] Ong MG, Lee PC, Tai CT, Lin YJ, Lee KT, Tsao HM (2006). Coronary Sinus Morphology in Different Types of Supraventricular Tachycardias. J Interv Card Electrophysiol.

[10] Ezhumalai B, Satheesh S, Anantha A, Pakkirisamy G, Balachander J, Selvaraj RJ (2014). Coronary Sinus Diameter by Echocardiography to Differentiate Atrioventricular Nodal Reentrant Tachycardia from Atrioventricular Reentrant Tachycardia. Cardiol J.

[11] Weiss C, Cappato R, Willems S, Meinertz T, Kuck KH (1999). Prospective Evaluation of the Coronary Sinus Anatomy in Patients Undergoing Electrophysiologic Study. Clin Cardiol.

[12] Lyubarova R, Boden WE, Fein SA, Schulman-Marcus J, Torosoff M (2018). Successful Percutaneous Coronary İntervention Significantly İmproves Coronary Sinus Blood Flow as Assessed by Transthoracic Echocardiography. J Echocardiogr.

[13] Ejibishvili C, Kiladze M, Begashvili I, Grigolia G (2024). Correlatıon between Ejectıon Fractıon and Coronary Sınus Blood Flow durıng Off-Pump Coronary Artery Bypass Graftıng Surgery. Georgian Med News.

[14] Zheng XZ, Wu J, Zheng Q, Zha WZ (2016). Coronary Sinus Flow is Reduced and Recovered with Time in Viral Myocarditis Mimicking Acute Coronary Syndrome: A Transthoracic Doppler Echocardiographic Study. J Ultrasound Med.

[15] Bietenbeck M, Florian A, Shomanova Z, Meier C, Yilmaz A (2018). Reduced Global Myocardial Perfusion Reserve in DCM and HCM Patients Assessed by CMR-Based Velocity-Encoded Coronary Sinus flow Measurements and First-Pass Perfusion Imaging. Clin Res Cardiol.

[16] Wei GL, Zheng XZ, Chen KQ, Shi YY, Wang LY, Tan XY (2018). Coronary Sinus Flow is Reduced in Methamphetamine Abusers: A Transthoracic Echocardiographic Study. Int J Cardiovasc Imaging.

