# Supplementary material for: Avaliação do Fluxo e das Dimensões do Seio Coronário por Ecocardiografia Transtorácica em Pacientes com Taquicardia por Reentrada Nodal Atrioventricular
Source: Arq Bras Cardiol. 2026 Jun 22;123(6):e20250891. [Article in Portuguese] doi: 10.36660/abc.20250891 (PMC13399413; doi:10.36660/abc.20250891)
Supplement: Translation

# Translation: Assessment of Coronary Sinus Flow and Dimensions by Transthoracic Echocardiography in Patients with Atrioventricular Nodal Reentrant Tachycardia

**DOI:** 10.36660/abc.20250891i

**Authors:** Ali Duygu, Alkame Akgümüş, Ahmet Balun, Fahri Er

**Affiliations:** 1 Bandirma Türkiye Bandirma Onyedi Eylul Universitesi – Department of Cardiology, Bandirma – Türkiye

**Keywords:** Atrioventricular Nodal Reentry Tachycardia, Coronary Sinus, Doppler Echocardiography

## Abstract

**Background:**

The coronary sinus (CS) plays a pivotal role in cardiac venous drainage and is anatomically adjacent to the atrioventricular nodal region. Although structural enlargement of the CS has been described in patients with atrioventricular nodal reentrant tachycardia (AVNRT), coronary sinus flow (CSF) assessed by transthoracic echocardiography (TTE) has not previously been investigated in this population.

**Objective:**

To evaluate the relationship between AVNRT and CSF.

**Methods:**

This retrospective single-center study included 35 patients with AVNRT who underwent successful slow-pathway ablation and 34 age- and sex-matched healthy controls. All participants underwent comprehensive transthoracic echocardiography. CS ostial diameter, CS diameter, and CSF were measured using pulsed-wave Doppler echocardiography. CSF was calculated using standard geometric and Doppler-derived parameters. A p-value <0.05 was considered statistically significant.

**Results:**

Global left and right ventricular systolic and diastolic function parameters were similar between groups. Patients with AVNRT exhibited significantly larger CS ostial and CS diameters compared with controls (both p<0.001). Despite this enlargement, CSF was significantly reduced in the AVNRT group (460.8 ± 83.3 vs. 523.8 ± 90.1 mL/min, p=0.004), accompanied by a lower CS velocity–time integral. Notably, CSF indexed to left ventricular mass also remained significantly lower in AVNRT patients, indicating that reduced flow was independent of myocardial mass.

**Conclusion:**

Patients with AVNRT demonstrate a distinct coronary sinus hemodynamic profile characterized by CS enlargement and reduced CSF as assessed by transthoracic echocardiography. These findings suggest altered coronary venous hemodynamics in AVNRT and provide novel pathophysiological insights using a non-invasive imaging modality.

## Introduction

The coronary sinus (CS) is a key structure for cardiac venous drainage, channeling blood from the cardiac veins into the right atrium. Due to its proximity to the atrioventricular (AV) node, it plays a critical role in electrophysiological procedures and ablation therapies. Atrioventricular nodal reentrant tachycardia (AVNRT) is the most common form of paroxysmal supraventricular tachycardia (SVT). It is caused by a reentrant circuit involving dual pathways located around the AV node, particularly within the Koch triangle – an anatomical area defined by the CS ostium (CSo), the tendon of Todaro, and the tricuspid valve annulus.^1^ Although the exact mechanism is unclear, studies suggest that AVNRT is related to a re-entrant circuit between slow and fast pathways located in the peri-CSo region.^2^ AVNRT ablation also occurs near the CSo within the Koch triangle. Previous studies have shown that the diameter of the CSo is larger in AVNRT patients, potentially altering conduction pathways and contributing to arrhythmogenesis. However, the role of coronary sinus flow (CSF), a measurable index of myocardial perfusion, has not been previously investigated in the context of AVNRT.^3-5^

In this study, we sought to explore the association between AVNRT and coronary sinus flow using transthoracic echocardiography (TTE).

## Method

The study was conducted with the approval of the Ethics Committee for Non-Interventional Research in Health Sciences at Bandırma Onyedi Eylül University (Date: 06.02.2025, Number: E-67961857-050.04-2500011721). All procedures were carried out in accordance with ethical guidelines and the principles of the Helsinki Declaration.

### Study design and population

This was a retrospective, single-center study including 35 consecutive patients diagnosed with AVNRT and 34 age- and sex-matched healthy controls. All patients in the AVNRT group underwent successful slow-pathway catheter ablation.

Patients with left ventricular (LV) systolic dysfunction, obstructive sleep apnoea syndrome, a history of medication or substance use, chronic kidney or liver failure, atrial fibrillation, congenital heart disease, a history of prosthetic valves, an aortic root diameter greater than 40 mm, a bicuspid aortic valve or a poor echocardiographic window were excluded. Patients with a history of coronary artery bypass grafting (CABG), acute coronary syndrome (ACS), ischemic stroke, portal hypertension, being under 18 years of age, connective tissue disease, advanced valve insufficiency or stenosis, right heart failure, a diagnosis of pulmonary hypertension, or a history of pulmonary embolism were excluded from the study.

Demographic, clinical and laboratory data were obtained from hospital records.

### Electrophysiological procedure

Catheters were used as percutaneous access devices and positioned using the Seldinger technique, with fluoroscopy employed to visualize catheter advancement. For CS cannulation, either an inferior or superior approach was used (6F steerable decapolar catheter, 2.52 mm, 110 cm, Boston Scientific, Massachusetts, USA). Two quadripolar catheters were placed in the right ventricle and in the His bundle region. A multichannel electrophysiology recorder was used to obtain a 12-lead surface ECG and intracardiac electrograms.

Each patient with SVT underwent a standard electrophysiology study, which included atrial and ventricular extrastimulus testing as well as overdrive pacing to induce tachycardia and determine its mechanism.^6^ The diagnosis of AVNRT was established according to standard criteria. After confirming AVNRT, slow-pathway modification was performed under fluoroscopic and/or three-dimensional mapping guidance. The inferior Koch triangle was identified as the target region for slow-pathway ablation.

### Echocardiography

Two-dimensional TTE (Vivid T8, GE Vingmed, USA) was used for all measurements. Echocardiographic assessments were performed with patients in the left lateral decubitus position. Left ventricular ejection fraction (LVEF) was calculated using the modified Simpson method. Interventricular septal thickness (IVS), posterior wall thickness (PWT), left ventricular end-diastolic dimension (LVEDD), left ventricular end-systolic dimension (LVESD), and left atrial (LA) size were obtained from the parasternal long-axis (PLAX) view.

The CSo was visualized by modifying the PLAX and apical four-chamber views. Measurements were taken vertically from the point where the CSo opens into the right atrium (Figure 1). Mitral inflow E, A, E′, and A′ waves were measured using tissue Doppler imaging (TDI), and tricuspid annular plane systolic excursion (TAPSE) was obtained using M-mode in the apical four-chamber view.

**Figure 1:** View of coronary sinus ostium.

The apical four-chamber and PLAX views were adjusted to visualize the CS. CS diameter (CSd) was measured at its widest point during ventricular systole, leaving a 1-cm distance from the ostium (Figure 2). To assess CSF, the CSo was first identified by modifying the PLAX view. The Doppler cursor was aligned with the CSo so that the angle between the cursor axis and the CS axis was less than 30°. A pulsed-wave (PW) Doppler sample volume was then placed 3 mm inside the ostium, in a region minimally affected by atrial contractions. The ventricular velocity–time integral (VTI) was calculated by tracing the systolic and diastolic CS flow waves (Figure 3). Heart rate was recorded from the ECG at the time of image acquisition.

**Figure 2:** Measurement of coronary sinus diameter.

**Figure 3:** Measurement of coronary sinus velocity-time integral.

Coronary sinus flow (mL/min) was calculated using the following formula: [π × D^2^ / 4 × VTI × heart rate]. Here, D represents the maximum diameter of the coronary sinus, and π is the ratio of a circle's circumference to its diameter.

### Statistical analysis

The sample size was determined by convenience, including all consecutive patients who underwent AVNRT ablation during the study period and met the inclusion criteria. An equal number of control subjects were enrolled. The IBM SPSS Statistics 26 package was used for the statistical analysis. Categorical variables are presented as absolute and relative frequencies and continuous variables are presented as the mean ± standard deviation if they are normally distributed, or the median and interquartile range (IQR) if they are not.

Distribution normality of the variables was examined using visual (histogram and probability graphs) and analytic methods (Shapiro–Wilk test). Two independent groups with a normal distribution were compared using Student's t-test, while those without a normal distribution were compared using the Mann–Whitney U-test. The chi-square test was used to compare categorical variables. Statistical significance is defined as p-values less than 0.05.

## Results

A total of 69 participants were included (35 AVNRT patients and 34 controls). The mean age of the AVNRT group was 32.00 ± 6.01 years, and the mean age of the control group was 29.35 ± 5.73 years. Demographic and echocardiographic characteristics were similar between groups, with no significant differences in left ventricular ejection fraction, chamber dimensions, or TAPSE values.

Both CSo and CSd were significantly larger in the AVNRT group compared with controls. CSF and CS VTI were significantly lower in the AVNRT group, and these differences remained significant after indexing to left ventricular mass.

All echocardiographic measurements are summarized in Table 1 and Table 2.

**Table 1:** Demographic and clinical characteristics of the study patients

		AVNRT	Control	p value
**Age (year)**		32.00 ± 6.01	29.35 5.73	0.066^a^
**Gender, n (%)**				
	Male	17 (48%)	14 (41%)	0.779^b^
	Female	18 (52%)	20 (59%)	
**Height (cm)**		169.14 9.01	170.47 ± 8.70	0.536^a^
**Weight (kg)**		75.49 ± 10.74	71.53 ± 7.77	0.085^a^
**BMI (kg/m^2^)**		26.60 ± 4.73	24.68 ± 2.70	0.043^a^
**HR (beat/min)**		64.03 8.18	67.71 ± 8.39	0.070^a^

AVNRT: atrioventricular nodal reentrant tachycardia BMI: Body mass index, HR: Heart rate.

a Student's t test,

b Chi-square test.

**Table 2:** Echocardiographic parameters

Variable	AVNRT	Control	p value
**LVEF %**	60.97 ± 4.91	61.15 ± 4.45	0.877^a^
**TAPSE (mm)**	24.89 ± 3.11	26.26 ± 3.17	0.073^a^
**LVEDD (mm)**	47.14 ± 4.65	45.24 ± 4.23	0.080^a^
**LVESD (mm)**	31.69 ± 4.23	29.79 ± 4.40	0.074^a^
**IVS (mm)**	11.20 ± 1.18	10.79 ± 0.67	0.087^a^
**PW (mm)**	10.51 ± 1.06	10.18 ± 0.67	0.122^a^
**Mit E (m/s)**	0.91 ± 0.06	0.91 ± 0.14	0.858^a^
**e’ (m/s)**	0.15 ± 0.01	0.14 ± 0.02	0.107^a^
**Mit A (m/s)**	0.68 ± 0.07	0.67 ± 0.10	0.502^a^
**a’ (m/s)**	0.08 ± 0.01	0.10 ± 0.03	0.336^a^
**E/e’**	5.98 ± 0.95	6.26 ± 1.26	0.298^a^
**LVMass (g)**	189.78 ± 46.20	144.41 ± 39.01	**0.000**^a^
**VTI (cm)**	12.33 ± 1.75	19.24 ± 2.60	**0.000**^a^
**CSo (mm)**	1.19 ± 0.12	0.94 ± .13	**0.000**^a^
**CSd (mm)**	0.87 ± 0.08	0.72 ± 0.07	**0.000**^a^
**CSF (ml/min)**	460.75 ± 83.26	523.78 ± 90.06	0.004^a^
**CSF index (mL/min/gram)**	0.371 (0.277-0.539)	0.278 (0.226-0.357)	**0.000**^b^

LVEF: left ventricular ejection fraction; TAPSE: tricuspid annular plane systolic excursion; LVEDD: left ventricular end diastolic diameter; LVESD: left ventricular end systolic diameter; IVS: interventricular septum; PW: posterior wall; VTI: velocity-time integral; CSo: Coronary sinus ostium; CSd: Coronary sinus diameter; CSF: Coronary sinus flow.

a Student's t test,

b Mann-Whitney U-test.

## Discussion

This study is the first to investigate CSF in patients with AVNRT using transthoracic echocardiography. We demonstrated that CSF is significantly lower and the CS diameter significantly larger in patients with AVNRT compared with healthy controls. This reduction in flow occurs despite preserved global ventricular systolic and diastolic function and is accompanied by marked enlargement of both the CSo and CSd. Together, these findings describe a distinct coronary venous hemodynamic phenotype in patients with AVNRT.

AVNRT is the most common regular arrhythmia in humans. Several models have been proposed to explain its mechanism, considering the complex anatomy and electrophysiological properties of the AV node and surrounding atrial structures; however, the true AVNRT circuit remains incompletely understood. In recent years, multiple studies have sought to clarify its underlying mechanism. One study examined the relationship between the CS and AVNRT and found that ablation targeting the site of earliest retrograde atrial activation within the CS in patients with eccentric CS activation produced highly successful outcomes.^7,8^

Another study compared typical and atypical AVNRT patients and found that the CSo diameter was wider in patients with typical AVNRT.^9^ In a study by Ezhumalai et al.,^10^ the proximal and mid diameters of the CS were examined in patients with AVNRT and AVRT using TOE. It was observed that the CS was wider in the AVNRT group.^10^ However, Weiss et al.^11^ showed that, although the CS diameter was wider in AVNRT patients, there was no significant difference between the AVNRT and control groups.

CSo enlargement stretches the periosteal tissue, increasing the distance between the slow and fast pathways and leading to conduction in different directions.^8^ Additionally, a larger CS may predispose to different AV node physiology by separating atrial entries to the AV node or increasing anisotropic conduction.^5^

A key observation of the present study is that coronary sinus enlargement in AVNRT patients is not associated with increased coronary sinus flow. On the contrary, CSF and coronary sinus velocity–time integral were significantly lower in the AVNRT group. This combination of increased venous diameter and reduced effective flow suggests a pattern of coronary venous remodeling rather than simple flow augmentation. Such remodeling may reflect chronic alterations in peri-nodal atrial anatomy, local venous compliance, or altered atrial–venous coupling adjacent to the slow-pathway region, as previously hypothesized in electrophysiological and anatomical studies.^5,8^

Importantly, global cardiac structure and function were largely preserved in AVNRT patients. Left ventricular ejection fraction, diastolic filling indices, and right ventricular systolic function parameters were comparable between groups, indicating that reduced CSF was not attributable to overt ventricular dysfunction. Although left ventricular mass was higher in the AVNRT group, indexing coronary sinus flow to myocardial mass did not eliminate the observed difference, supporting the notion that reduced CSF represents a distinct hemodynamic feature rather than a secondary effect of myocardial hypertrophy.

CSF has previously been shown to reflect myocardial perfusion and global coronary circulation in various clinical conditions, including ischemic heart disease, myocarditis, cardiomyopathies, and following revascularization procedures.^12-15^ In addition, reduced CSF has been associated with non-coronary conditions such as substance-related myocardial injury.^16^ However, its role in arrhythmic substrates has not been investigated. The present findings suggest that altered coronary venous hemodynamics may coexist with the anatomical and electrophysiological substrate of AVNRT. While causality cannot be inferred from this observational study, reduced CSF may reflect chronic adaptations in the peri-nodal region that contribute to, or result from, the AVNRT substrate.

This study has several limitations. Its retrospective design and relatively small sample size limit causal inference. CSF was assessed at rest and at a single time point, and invasive hemodynamic measurements were not available for validation. Additionally, although meticulous Doppler alignment was performed, CSF measurement by transthoracic echocardiography remains technically challenging. Nevertheless, the consistency of findings across multiple coronary sinus–related parameters strengthens the validity of our observations.

## Conclusion

Patients with AVNRT exhibit a distinct coronary sinus hemodynamic profile characterized by coronary sinus enlargement and reduced coronary sinus flow as assessed by transthoracic echocardiography. These findings provide novel non-invasive insight into coronary venous hemodynamics in AVNRT and suggest that coronary sinus flow assessment may serve as a complementary echocardiographic parameter for understanding the pathophysiology of this arrhythmia.

## Data Availability

All datasets supporting the results of this study are available upon request from the corresponding author.
